# Readiness for Heart Failure Self-Care: Commitment and Capacity

**DOI:** 10.3390/healthcare13141725

**Published:** 2025-07-17

**Authors:** Stephanie L. Turrise, Carolyn Kleman, Caroline Jenkins, Nia D. Lewis, Heidi Winslow, Bridgette Williams, Kori E. Meyer, Sonya SooHoo, Barbara Lutz

**Affiliations:** 1 School of Nursing, College of Health and Human Services, University of North Carolina Wilmington, Wilmington, NC 28403, USA; klemanc@uncw.edu (C.K.); caj2596@uncw.edu (C.J.);; 2 Novant Health New Hanover Regional Medical Center, Wilmington, NC 28401, USAbridgette.williams@novanthealth.org (B.W.); 3 Department of Pyschology, College of Science and Engineering, University of North Carolina Wilmington, Wilmington, NC 28403, USA; 4 Independent Researcher, San Francisco, CA 94122, USA

**Keywords:** heart failure, self-care, health behavior

## Abstract

**Background**: The number of people with heart failure (HF) is rapidly increasing globally. Self-care plays a key role in improving HF outcomes. The readiness to engage in heart failure self-care (HFSC) behaviors encompasses a commitment to change and the capacity to make the change. Commitment is a personal investment and value toward enacting self-care and health-related behaviors. Capacity includes an individual’s skills, knowledge, beliefs, previous experience, and resources. **Aim**: The aim of this study was to describe patient-identified commitment and capacity factors influencing their readiness to carry out HFSC behaviors. **Methods**: A qualitative study using thematic analysis was conducted using data from 21 interviews to develop readiness for HFSC themes. **Results**: The commitment themes identified were cultural values and beliefs, social roles, will to live, attitude, self-efficacy, HF perceptions, and general emotional state. Capacity themes included HF literacy, functional capacity, environmental resources, comorbidities, time, cognitive functioning, and social support. **Conclusions**: Novel themes related to an individual’s commitment to HFSC activities included their will to live and social roles, while time emerged as a consideration in the capacity to engage in self-care. To optimize HF outcomes, people with HF must be ready to engage in HFSC. Evaluating an individual’s readiness for HFSC can focus healthcare team efforts on targeting specific self-care activities that require intervention. Enhancing readiness by intervening with specific commitment and capacity factors is a step toward optimizing HFSC and improving patient outcomes.

## 1. Introduction

Heart failure (HF) is a complex and progressive condition affecting 56.5 million people globally, with 6.7 million individuals in the United States and numbers growing worldwide [1,2]. Self-care is a key component in preventing and managing HF [3]. Once diagnosed with HF, people must change their lifestyle and daily behaviors to engage in heart failure self-care (HFSC). Individuals with HF find making these changes challenging [4]. Two theoretical models consider HFSC and change. The revised Situation Specific Theory of Self-Care [5] describes how situational characteristics (person, problem, environment) link to the natural decision-making process, which is influenced by experience, knowledge, skills, values, and symptom perception that direct HFSC actions. The Transtheoretical Model (TTM), also known as the Stages of Change, is an integrative model that conceptualizes the process of individual behavioral change [6,7,8]. There are five stages, ranging from pre-contemplation, where an individual is not ready to change, through maintenance, when the behavioral change has been maintained for at least six months. While the TTM can help identify an individual’s commitment to engage in a behavior change, it does not help identify their capacity or capability to engage in that change. Additionally, the TTM does not address social determinants of health or the context within which changes must occur and directly impact individuals with HF. Another important limitation of the TTM is that it does not consider multiple, complex, life-long changes, such as those required in chronic illness, in general, and HF specifically. Finally, the TTM assumes that individuals make rational, coherent, and logical decisions about behavior change, which is not always true.

Commitment and capacity are two crucial components used to define the concept of readiness [9,10]. Readiness is not a new concept and is identified as being a critical factor in successful organizational change. Organizational readiness includes a commitment to change and the capability to make the change [10]. When readiness is not assessed, moving forward with the change can create a crisis [11]. However, we do not apply this same ideal to individual behavior change. In current practice, individual readiness to engage in HFSC or readiness to change lifestyles and health behaviors is not assessed, even when the needed changes are large and complex due to multiple comorbidities commonly present in people with HF [2]. Instead, healthcare professionals (HCPs) often expect that if someone has a life-changing event, such as a HF diagnosis, that person understands the significance of the diagnosis and will be motivated to make all the necessary changes. 

During the past two decades, healthcare has shifted from a paternalistic approach, where people are expected to follow “orders,” to a person-centered approach, where shared decision-making models are implemented, and HCPs work with people to enhance their engagement in self-care. However, current practices assume that individuals are ready (i.e., have the commitment and capacity) to engage in HFSC without a systematic assessment of readiness. There is limited research on readiness to change in people with HF. Only one study related to readiness in HFSC was identified [12]. Using the TTM, the study aimed to identify the stage of readiness for change with six lifestyle behaviors important in HF to determine differences in HF signs and symptoms, self-reported HF knowledge, and self-care behavior between patients who acted and patients who did not. The authors found that using the stages of change tool to assess readiness to make lifestyle changes was insufficient to assess readiness in patients with HF, perhaps because of the number and complexity of the needed changes. Lutz and colleagues [10] identified the concept of readiness to engage in stroke caregiving and developed a theoretical model that included commitment and capacity readiness domains. While a caregiver may be committed to providing care, they may not have the capacity to do so [10]. However, commitment without capacity does not allow for successful implementation of disease management strategies. For example, individuals may be committed to weighing themselves every day, but without the scale to do so, they cannot monitor critical changes in weight. The reverse is also true, having a scale but not being committed to daily weighing results in the same outcome. Commitment is essential for managing chronic disease and is defined as a personal investment and value toward enacting self-care and health-related behaviors [13]. Both commitment and capacity are needed for effective HFSC. Capacity includes internal and external resources and experience to engage in the desired behavior. Based on the theoretical and empirical evidence, we hypothesize that factors related to commitment and capacity, combined with elements in the HF literature about successes and challenges in engaging in HFSC, comprise readiness, a necessary precursor to successful HFSC.

People with HF must commit or agree to many behavioral changes in the maintenance phase, such as taking medications, weighing daily, and reducing dietary sodium. Capacity includes an individual’s skills, knowledge, beliefs, previous experience, and resources, e.g., finances, social support, and access to community services. An individual who may be committed, but lacks capacity, for example, because they do not have sufficient resources, such as funds to buy medications or equipment, will have difficulty successfully engaging in self-care behaviors.

Personal characteristics related to HFSC are internal factors that can influence behavior. A search of the literature shows that symptom severity, experience, skills, motivation, habits, education, cultural beliefs and values, functional and cognitive abilities, confidence, attitudes, symptom recognition, excessive daytime sleepiness, depression, anxiety, and the number of comorbidities were all personal characteristics that influence HFSC behaviors [14,15,16,17,18]. Environmental characteristics related to HFSC are external factors in the patient’s environment that can influence behavior. Social support and access to care have been identified as environmental characteristics that influence HFSC behaviors [19,20,21,22]. There is overwhelmingly more evidence identifying the personal characteristics that influence HFSC, and there is some knowledge of environmental aspects, but little is known about systemic factors [21]. All levels of factors are critical to an individual’s readiness for HFSC. 

The purpose of this study was to define readiness for HFSC by considering current evidence and exploring individual, environmental, and systemic factors that influence readiness to engage in HFSC. The specific aims of the proposed study were to confirm already identified individual factors [21], uncover any novel individual factors, and explore environmental and systemic factors.

## 2. Materials and Methods

### 2.1. Procedures

An inductive qualitative approach with applied thematic analysis was employed to explore the self-care experiences of people with HF, focusing on their readiness to perform necessary HFSC behaviors. Applied thematic analysis helps identify ideas and themes in textual data while ensuring that these interpretations are grounded in the data itself, rather than being imposed by the researchers. Applied thematic analysis is a rigorous approach using set procedures to identify themes in a transparent, credible, and reproducible way [23]. Recruitment site institutional review board approval was obtained (IRB #23-2378) and all participants provided written informed consent before interviews were conducted.

### 2.2. Sampling

Purposive sampling ensured the recruitment of participants who were currently self-managing HF. Participants were recruited from a local HF clinic and a level two trauma center (re-admissions). Potential participants were identified daily in consultation with the clinic and hospital staff. Individuals were included if they had a HF diagnosis, were 18 years or older, English speaking, and lived at home. Individuals were excluded if they had cognitive impairment (score of 9 or higher on the Short Blessed Test [SBT]) [24], which could interfere with HFSC abilities and ability to consent. One individual scored above nine on the SBT and was excluded. Those who met the inclusion criteria were approached by an investigator who explained the study, including the gift card incentive, invited them to participate, and gave them a copy of the consent to review. If individuals met the inclusion criteria, their contact information was collected, and a time was set up for the interview in their location of choice. There were no dropouts once individuals were interviewed, but some people changed their minds when called to schedule the interview due to the timing of the interview (i.e., they were traveling, family visiting, or not available in the timeframe), with two people not returning phone calls. In the hospital re-admission study used for the secondary data analysis, only one person was lost to follow-up between initial recruitment and calling to arrange the interview.

### 2.3. Data Collection and Interviews

The team conducted 11 qualitative interviews with people with HF to learn about their experiences and what it means to be ready to engage in self-care. Additionally, 10 previous interviews with people who had HF and were re-admitted for a HF exacerbation [25] (for more information) were used for secondary data analysis. These interviews were included because while the parent study was not focused on readiness, this theme emerged during the interviews and was the impetus to undertake the current investigation. Thus, a total of 21 participants were interviewed. Once interviews were scheduled, the PI conducted all interviews with one other team member in all but two instances, where it was just the PI due to the availability of team members. All but one interview, which was conducted on campus, was completed where the individual was living. Participants were reminded about their rights as research participants, questions answered, and written consent obtained. During the interviews, there were occasionally families, or significant others present who participants wished to be part of the interview and also signed consent. Interviews were audio recorded and professionally transcribed. Interviews in the clinic group ranged in length between 19.5 min to 71 min with a mean of 45.5 min and in the re-admission group ranged between 25 and 75 min, with an average length of 43 min. The interviews were recorded using a digital voice recorder, downloaded as an audio file to a secure password-protected computer for safe storage, and professionally transcribed with all identifying information, such as names and places, removed. Interviews were loosely structured with the interview questions and probes guiding the conversation. Sample questions from the interview guide included: (1) What does being ready to take care of yourself mean to you?; (2) Based on your experiences and what you have learned caring for yourself and your heart failure, what are the key things you think must be done to manage your heart failure? and (3) Can you tell me about your commitment to taking care of yourself, your heart failure, and your health? The interview guide from the re-admission group was focused on the experience of a heart failure re-admission with questions that included: (1) Tell me about your heart condition.; (2) Tell me about your recent hospitalization for your heart condition. What was that like for you?; and (3) Is there anything else you would like to tell me about your experiences with your heart condition or your recent hospitalization? The participants were asked to speak about their own thoughts, feelings, experiences, and perceptions, and encouraged that there were no right or wrong answers to promote authentic responses and enhance credibility. Finally, the participants completed general Likert scale questions about HFSC. Participants received a USD $40 gift card for compensation of their time.

The investigators have a history of research in identifying factors impacting self-care in HF and vascular disease and self-care [9,21,25]. There were no identified personal characteristics that may have influenced the research, and researchers had no formal (caregiving) or informal relationship with the participants. Assumptions based on preexisting knowledge informed the interview protocol design.

### 2.4. Data Analysis

Ongoing, concurrent, and comparative data analysis was carried out. As transcriptions were completed, data were analyzed in two ways. Transcripts were first reviewed and validated (CJ and KM) against the audio recordings. Two researchers on the team (CK, ST) reviewed and coded the transcripts, looking for commonalities and differences among the data within and across interviews using the lens of the current study, readiness for HFSC as the focus for the secondary analysis. Coding consisted of identifying similar phrases, patterns, ideas, sentiments, themes, sequences, and key features in the data. Initial coding was conducted independently and then codes were reviewed together (CK, ST) to identify any discrepancies or differences, which were discussed until a consensus was reached. Concurrently, the rest of the team was reading and reviewing transcripts to familiarize themselves with the data. The themes began to repeat, indicating data saturation, with the eighth interview. Three additional interviews were conducted to verify preliminary themes, with a focus on recruiting females and those with a more recent HF diagnosis, ensuring no additional or different themes or information emerged. With coding completed, the codes were then examined to determine if some broader categories or themes could be derived from the data and compared to existing knowledge and theories. The coding structure and themes were then shared with the larger research team to discuss and deliberate. Additional clarification and review of transcripts were conducted to reach consensus.

The study’s rigor was maintained using such strategies as purposeful sampling, accurate transcription of the data, and establishing rapport with the participants and their family members to enhance honesty and openness during the interviews. Qualitative data collection procedures were recorded and reported in detail to strengthen transferability. The interview guide with probe questions was developed by three of the authors (CK, BL, and ST). The order of questions was revised after the eighth interview to enhance credibility, ensure participants were not led by the question order, and verify that the guide covered key topic areas, including. exploring the emerging concept of culture. Four of the authors (CJ, CK, BW, and ST) have extensive experience working with people who have HF, and one author is an expert in qualitative research (BL), which further enhances credibility. Journaling was completed after each interview by the PI as a form of field notes to aid in constructing context, encounter, and interview data [26]. Member checking was also conducted with five participants upon the conclusion of coding, sharing themes and sub-themes to validate that they captured the essence of the interviews.

## 3. Results

Participants were majority male, White, with ages ranging from 47 to 92 years old representing seven counties in Southeastern North Carolina. All participant’s selected gender was the same as their sex. The study sample characteristics are listed below in Table 1.

### 3.1. Themes

Several themes emerged from the interviews and were further categorized based on their fit with the two prior readiness concepts, commitment and capacity. Commitment themes included cultural values and beliefs, social roles, will to live, attitude, general emotional state, HF self-efficacy, and HF perceptions. Capacity themes included HF literacy, functional capacity, environmental resources, comorbidities, time, cognitive functioning, and social support.

#### 3.1.1. Commitment Themes

**Theme I: Culture values and beliefs.** This theme is defined as the principles, assertions, and ways of life that influence the thinking, decision-making, and actions that individuals make when completing their HFSC activities, which may include influences from the social, spiritual faith, geographic, and other dimensions. When asked what guides her HFSC one participant responded “Prayer. Just thanking God, because I’m still here. Thanking God because I get to see my kids every day, and just my faith, all around, because I’ve been through a lot throughout my lifetime” (P2). Some participants specifically identified cultural practices that influenced their dietary choices and habits; for example, one individual stated, “I’ve learned from self-experience diet. Especially, you know, being Black for the simple reason we season our food heavy. Our mama did, our grandma did, our great grandma did” (P8). Other ways in which culture influenced HFSC included beliefs about healthcare seeking. Two participants suggested they rarely sought care unless it was absolutely necessary, they needed to tough it out. One participant said, “Keep from having to go to jail, but as far as being sick, I would keep—I would just like colds or whatever. You know, I’d stay home or stay out of the way or whatever. Going to the doctor won’t in me. I mean, I—I ain’t got no business there” (P12) and another said, “I ain’t the type to want to go to the hospital. I mean, I’ve been cut with chainsaws and put Krazy Glue on it…. —if—if you could stop the bleeding we didn’t go to the doctor” (P6).

**Theme II: Social roles.** Social roles are defined as *providing social support* to family, friends, and others; valuing social roles and responsibilities; and fulfilling the responsibilities of daily living to others. When discussing the importance of HFSC, one person noted that she is motivated by her role as a mother, stating the following: “Yeah, and it’s like I can’t go through this again. I can’t go through this. My daughter’s only four. My baby’s only four. I can’t, I have to be here” (P2). Another said “My son relies on me” as he became very emotional discussing his need to be healthy and well to care for his son (P9). When asked how long it took to learn the importance of engaging in HFSC, another participant indicated “It was immediate cause I knew I had to still function and take care of my family and live and work” (P5).

**Theme III: Will to live.** The will to live is defined as one’s expression of commitment to life, their desire to continue living, and the feeling that they still have more to do or accomplish in this life. Every participant mentioned the will to live either as a reason to do HFSC, or as a factor in their decision not to engage in HFSC recommendations. For instance, one woman who had multiple hospital admissions stated “I’m 72 years old. I don’t intend to change a thing at my age now and I don’t” while giving the rationale that she had outlived all her family members, “I’ve lived to be older than any of ‘em... I’m doin’ somethin’ right” (P4). When asked what motivates them to perform HFSC, one participant said, “I wanted to live a-a little bit longer, you know” (P1). Another described his motivation to engage in HFSC by stating, “I wanna live. Oh, yeah. I-I ain’t sayin’ this cynically, but I d—I-I guess—I just wanna stay here, you know what I mean, that’s all” (P7) while another stated, “Because I got sick, and I wanted to live. I didn’t want to die yet. I feel like I got a few more things to do” (P11). The will to live was pervasive in the discussions as others said, “Uh, that I wanna stay on this side. I got-I got a thrill to live, and-and I feel I’m blessed even to be here, you know? I know people that have had it that—that’s not here now (P7), “Nah, nah, I’m not gonna die this week. I just wanna be here” (P10).

**Theme IV: Attitude.** General evaluation of an individual’s view of life, health, and HF. When asked about how HF has impacted his life, one individual said, “Somebody could be worse” (P5). Another participant Patient reported attempting to make lifestyle changes to improve his HF management, “But I intend to cut down on my meals, and I’ll take my medicine and try to get in some exercise every day or two. I’ll stay off of—get off the salt, which I’m working on. And, uh, that’s what’s in that bag, no-salt stuff” (P11).

**Theme V: HF Self-efficacy.** HF self-efficacy can be defined as a belief in one’s ability to perform and manage their HFSC activities including problem-solving and troubleshooting deviations from their norm/baseline. When asked if they had any advice on HFSC, one participant stated, “Uh, learn all about your disease you can. You know, educate yourself. Ask the tough questions. You know, if you don’t think the doctor is doing like he wanted to or was supposed to or think he should do, ask him why. Don’t be—I used to be scared to ask the doctor anything, but now I’m not” (P20). To manage HFSC another participant encouraged others to “Read the paperwork, do your research, know your medications and how your body reacts to them” (P1).

**Theme VI: HF Perceptions**. HF perceptions include a person’s cognitive appraisal and personal beliefs and perceptions of HF, how one experiences physical and emotional symptoms, controls, and frames living with HF. When asked about engaging in HFSC, one individual reported, “I ain’t as stubborn as I used to be and, um, I know I ain’t doing like the doctors tell me to do, far as my diet and exercising and, you know, stuff like that” (P6). Another individual indicated his perceptions are shaped by how the healthcare team members lead the care, “It’s something—you don’t know if it’s serious or not if nobody tell—don’t tell you” (P11). When describing how HF has impacted his life, another stated, “Just don’t let it get you down. I mean, there’s just been a lot of times I’ve sat here and just felt like I was gonna cry because, you know, I look at my life five years ago, and I look at it now. There’s a big difference” (P20). One participant indicated that he has acquired an expertise on his HF and his symptoms saying, “You know, I’m kinda more—I’m kind of experienced about it. You know, really I am. But, you know, I ain’t, you know, trying to second guess them, but still, that’s my opinion” (P20). One participant described understanding how emotions can impact her HF symptoms, but even with understanding, there are other emotions about worsening HF, “That comes with the anxiety. I already know that. It was a anxiety thing, and if it’s my heart again, and I get scared” (P2).

**Theme VII: General emotional state.** General emotional state includes subjective experiences and interpretations, as well as feelings of negative emotional states. When asked what makes it harder to engage in HFSC, one individual said, “Sometimes you get depressed. I been going to mental health for 20, going on 20 years this year” (P1). Another responded that the HF impacting his physical functioning has caused emotional distress, “…and that’s what worries me out about laying here. I can’t stand it. It tears my nerves up” (P6), while another participant stated, “You get a little down. And you feel like, you know, um, your body is just not gonna kick back in. And—and went through a little down stage there” (P18). Stress was another negative emotion cited by participants, “But stuff like that keeps stress on ya. And I can—and when there’s a lot of stress, then I stay sicker” (P6).

#### 3.1.2. Capacity Themes

**Theme I: HF literacy.** This theme is defined as one’s awareness and understanding of what HF is, its management (medical and self-management), and its trajectory that includes knowledge seeking, access, and use to make decisions and take action. When asked how she obtains information about HF, one participant reported,

“I read, not just one, not just two. I’ll probably read three or four, just to see, to compare the information, because if it’s different on all of them, look, I’m going to the doctor. I gotta call the doctor, or the nurse line. I call the nurse line. I don’t know something and I wanna know, it’s the nurseline” (P2).

One participant described the challenges in his HF understanding,

“I mean, they said, ‘Well, you got congestive heart failure.’ I got what. I mean, I’m just old country boy. Ain’t ever learned a whole lot, but what did that do? And well, which Dr. X and the nurses, people tried to explain it to me where I can understand it” (P12).

Another individual described learning about HF as overwhelming, stating, “Oh, tons of information. It was overwhelming. And then, you know, when you get on the internet, you hit the wrong button—and you see, [whispered] “You’re gonna die” (P5). Another participant also suggested that while she is confident and capable of finding reliable HF information, she also mentioned the negativity that can come with reading about the HF illness trajectory, “I look at more than one source. I don’t just accept the first thing I see. Really, I don’t spend a lot of time researching it ’cause I don’t wanna psych my own self out” (P5).

**Theme II: Functional capacity.** Functional capacity is an individual’s ability to complete physical tasks, which reflects their aerobic capacity, endurance, and tolerance to activity, and can be measured via different evaluations such as a 6 min walk test, ejection fraction, and NYHA classification. When asked how functional capacity affects HFSC, one person reported needing assistance, “Now, um, a lot of the times when I go, uh, shopping sometimes I have to get my boys to go with me, or I have been in a grocery store and had to find somewhere to sit down. I couldn’t go no further. Um, I ask to push the buggy cuz that’s why—it’s like a crutch or something, you know” (P6). Another’s partner reported exercise is difficult stating, “But he’s tryin.’ You know, he’s—he gets out there, and he tries. Um, but they said that exercise would be a lot more beneficial if he can do it” (P18). A third participant also indicated how HF fatigue has impacted her ability to do HFSC activities she knows she must do, “But it’s been harder on me because of my heart. And I don’t have the energy to do it” (P13).

**Theme III: Environmental resources.** The places, spaces, and conditions in which an individual lives, works, and recreates, as well as the resources available for HFSC and health, define the theme of environmental resources. One individual indicated he had to make living accommodations due to his HF, stating “ I’ve had it since I—I’ve been here seven years, and I had it before I come here ’cause my doctor told me I had to move from where I was stayin’ ’cause I had to climb steps to get to my apartment” (P7). Another common sentiment related to resources was having someone to talk to about their illness, the information they received, and the feelings they were having. One individual said the following:

“….some things you wanna get out, and, you know, you know, you hold a lot of stuff back in when it’s just you, ’cause I’m a single man. And you-you hold a lot of stuff in. You don’t have nobody to share it with, so you hold it in, but when somebody—you get the chance to do something, you—hey, let it fly” (P7).

Two additional participants expressed similar thoughts about talking things through with others,

“Yeah. I-I think it would be. I think it would be helpful. You know, somebody knowledgeable there, uh, and had some understanding of what was happening, that I felt very comfortable. That they’re not just here to hear the medical part, but they’re here to hear the psychological part too and that’s, uh, what they’re trained for….” (P3).

While this previous participant was referring to a professional, others sought this type of assistance from individuals close to them such as friends or family members.

“That is, um, kind of the way I feel about that. I’ve never—but I do have a friend that once in a while she and I can get together and talk. But she has her own problems too. So therefore, you know, sometimes we take turns. You tell me your problems, I tell you my problems. You know, we talk it over and do things. And by the end of the day, we think huh, we’re okay now” (P13).

**Theme IV: Comorbidities.** Comorbidities are medical conditions, acute or chronic, that coexist alongside one’s HF and affect an individual’s health, treatment, self-management, and prognosis. When asked about other conditions that affect HFSC, one gentleman reported “I stay in AFib a lot and they can’t figure it out” (P6) while another participant’s spouse described multiple comorbidities making care HFSC more challenging stating “He’s got—he’s got, um, diabetes. He’s got high blood pressure. He’s got all of this stuff associated with Agent Orange. So, um, you know, he’s got a lot of issues” (P18). Another indicated that her comorbidities contribute to attending to and identifying symptoms and their causes, “…all of a sudden, you can barely stand up, not for too long. If I stand up too—even washing the dishes, then I get all this pain shooting up my back. What is that? Okay, is that the RA, neuralgia, or what is that?” (P2). Another participant reported, “And I was feeling good ‘til I got a hernia. It threw me in the hospital, and it just loaded me down with fluid” (P20).

**Theme V: Time.** Time was an important and novel theme identified across interviews and is defined as the amount of time an individual has available to dedicate to the management of their health, including HFSC activities. A participant and his spouse detail the importance of having time to weigh each morning, “But you’ve got to catch it before it gets too bad because once it gets to the point that it’s affectin’ his breathin’ then I can’t do anything to correct that. I can’t handle that. But now we weigh—he weighs every morning. And if there’s an accumulation of two and a half to three pounds plus that imprint of the thumb, I know he needs to start pulling off—we need to pull off extra fluid” (P18). Another participant suggested that things have gotten easier for HFSC since her retirement and her caregiving responsibilities have diminished, stating “Yeah because at the time that I was diagnosed, I think my mother was still living. I was her caretaker. I was in grad school, and I was working full-time. This gives me a lot of time just to focus on me” (P5).

**Theme VI: Cognitive functioning.** An individual’s memory, attention, and executive functioning reflect their ability to make decisions, plan, and problem-solve, which are required for an individual to complete HFSC tasks. When asked how their cognitive functioning impacts HFSC, individuals indicated such impacts as, “Had a little memory loss….I forget the days of the week….” (P1) and “Sometime my memory is not that great. ‘Cause I forget” (P18). Another participant indicated “And still, there’s certain memory gaps” (P3). Participants detailed strategies they use to deal with cognitive impairment reporting, “That’s me. I write it down” (P18), and another indicated “document things…I got a tablet….’Cause if I don’t I forget. Goin’ to the doctor on the right time or to the call” (P1).

**Theme VII: Social support.** The perception that an individual is cared for and *receives or has available* tangible and intangible assistance from other people, either formally or informally, and can be emotional, informational, or instrumental. One individual reported social support is crucial, “I think that’s real important for—for the family to get involved, you know. To get involved enough that they know what’s goin’ on” (P18). Another participant credits his partner with him being alive stating, “Well, if she wasn’t here, I’d be dead. Yeah. I would just—what I’d say the other day, I’d be out on the porch having a smoke, drinking a beer, and cussing the neighbor, and fall over dead” (P17). A spouse who participated in interviews noted the following about family and the support their children gave to their father:

“But, uh, we’ve got four daughters that are—I guess they’re about the best medicine that anybody could take because they’ve got him rotten. They come in and they were takin’ turns comin’ in, and they were makin’ sure Daddy was gonna be all right. And they were tellin’ him how he better—they sayin’ around here now is tie a knot and hold on.

So they were makin’ sure that he held onto that knot that they had tied for him, so, um, they tell him right quick. “No, Daddy. You’re not—you’re not a quitter. You’re a fighter” (P18).

Other participants had social support that provided them with key resources when needed as one participant described his sister, “She done keep me outta—she took me in, so she want me—she-she don’t want to be the one walk behind me in a black dress, cry. So she told me to live with her a while” (P10).

## 4. Discussion

**Figure 1:**
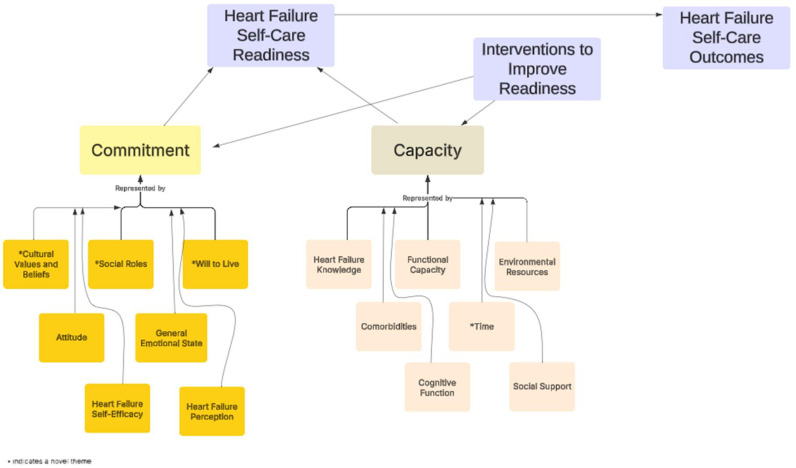
The proposed model of readiness to engage in heart failure self-care.

A total of 14 themes defined readiness for HFSC (See Figure 1). Novel themes related to commitment were the will to live, social roles, and cultural values and beliefs. Social roles include the social support that the HF participant *gives*. In contrast, social support in capacity is about the perceived social support the participant or person with HF *receives*. Considering the social support someone gives is unusual to measure, yet is clearly an important motivator to engage in HFSC. Patients are largely motivated by personal obligations and duties to serve others, which affects their readiness to engage in HFSC [27]. Chew and colleagues [28] also found that motivation could be enhanced by encouraging individuals with HF to consider their family’s future well-being.

The will to live was mentioned by all 21 participants and served as an important factor in the individual’s commitment to engage or not to engage in HFSC. The will to live can present as feeling discouraged due to the diagnosis or as a call to action or wake up call to engage in self-care to satisfy personal interests and obtain physical autonomy [29]. Social roles also appeared to affect an individual’s will to live. Participants expressed a need to live for the family members who are even more vulnerable than they are and depend on their support as a caregiver. Paturzo and colleagues [29] reported that family was a source of the will to live in many participants, which can be a positive factor. However, it also impacts time, one of the capacity factors. Being a primary caregiver for a family member can result in increased workload, stress, and burnout, negatively impacting an individuals’ readiness to engage in HFSC [30] or at least make it more complicated to manage.

Cultural values and beliefs, including religion, geographical region, and cultural practices heavily influence HF patients’ diet. The habits and practices of religions and cultures can also influence the commitment and motivation to perform HFSC activities. Cultural and spiritual values are crucial in shaping HFSC management [31]. Incorporating the cultural framework in the components of interventions can improve adherence to HFSC activities [27]. Understanding unique lifestyles, traditions, and dietary habits allows interventions to be tailored to meet each individual’s specific needs while respecting, incorporating, and altering necessary cultural practices to be more HF friendly [31].

The theme of time was a novel theme for capacity to perform HFSC. Time available to perform HFSC is affected by social roles and responsibilities, including the time needed to provide caregiving for others, which was reported by all age groups in this sample. Kleman and colleagues [32] in a focus group study on the work of managing a chronic illness indicate that time is a resource needed for the “job” of managing a chronic illness. Additionally, people with other comorbid conditions have self-care activities they must perform for other comorbid conditions, influencing the time they can devote to HFSC. Having or making the time to dedicate to HFSC requires one to identify, plan, and monitor plans, amending and revising plans as needed, and having forethought in some instances, such as when traveling, which requires a high level of executive functioning, which can be affected by impaired cognitive function. Time constraints are also compounded by functional capacity. Functional capacity is a necessary consideration in relation to time because it affects how much time it takes an individual to perform self-care activities. There are elements of time outside the patient’s ability to control that impact the ability to perform HFSC, which include the time it takes to contact and receive a response from a provider, administrative work needed to complete the activity, and time for other activities of life. Functional capacity, social roles, and comorbidities have a compounded effect on the time an individual must devote to HFSC.

## 5. Practice Implications and Recommendations

To provide patient centered care a holistic view of readiness for HFSC needs to be considered and adopted. Findings indicate that HCPs must address both commitment and capacity factors to improve readiness for HFSC. These factors, such as social roles and responsibilities, and time for HFSC activities, are interconnected. Addressing only one factor, through a social determinants of health questionnaire, for instance, may not fully support the behavioral changes needed for optimal HFSC. Although outpatient healthcare entities are not required to evaluate capacity via SDOH screening, it should be considered. Having a holistic framework or model to guide screening can contribute to care efficiency, consistency, and promote multidisciplinary collaboration. A specific screening tool for HF that includes both capacity and commitment factors could provide actionable insights to direct targeted interventions that optimize HFSC, decreasing unnecessary or overlapping resources and services, and enhancing patient readiness for optimized HFSC. At a minimum, healthcare professionals should incorporate these concepts into their encounters to initiate and maintain dialog, adapting as the individual’s condition and needs change. Patient–provider communications that consider and focus on cultural values and beliefs, social roles, the will to live, time constraints, and the other factors identified could improve readiness and accelerate action. Utilizing these themes as the framework for assessing readiness in other chronic illnesses requiring intense lifestyle and behavioral changes is recommended to test for the wider applicability and generalizability of the readiness themes identified in this study.

## 6. Limitations

The themes identified in this study were from interviews with 21 participants with HF in Southeastern North Carolina and may not be generalizable to broader populations in other geographic locations. Interviews were conducted during two different time periods and settings—inpatient and outpatient—which could limit the generalizability of the findings. This study focused on the participants’ readiness to engage in HFSC and did not assess caregiver readiness, which emerged as a consideration in promoting HFSC via social support. Additional research is needed to explore caregiver readiness in HFSC.

## 7. Conclusions

Readiness for HFSC is grounded in commitment and capacity factors that reflect individual, family, community, and systems readiness. There is a need for assessments that screen for commitment and capacity factors so they can be addressed to enhance readiness to engage in HFSC. Evaluating readiness for HFSC will focus care team efforts on matching actual needs with resources and behavioral interventions targeting specific self-care activities to improve HFSC.

## Figures and Tables

**Table 1:** Sample Demographic Characteristics.

Demographic Characteristic	Total n = 21	Percentage
Age in years		
Range	47–92	NA
Mean	64.7 years	NA
Sex		
Male	15	71%
Female	6	29%
Race		
African American	9	43%
American Indian	1	5%
Caucasian	11	52%
Ethnicity		
Hispanic or Latino	0	0
Not Hispanic or Latino	21	100%
Education		
Master’s/Professional	4	19%
Associate or Bachelor’s degree	7	33%
Some college	4	19%
High school graduate or less	6	29%
Months with illness		
Mean	67	NA
Range	2–216	NA
Employment status		
Retired	12	57%
Disabled, unable to work	8	38%
Prefer not to answer	1	5%
Annual income		
USD 25,000 or less	7	33%
USD 25,001–USD 50,000	3	14%
USD 50,001–USD 75,000	1	5%
USD 75,001–USD 100,000	2	9%
over USD 100,000	2	10%
Prefer not to answer	6	29%
Insurance		
Private	3	14%
Public	12	57%
Both	4	19%
Uninsured	1	5%
Prefer not to answer	1	5%
Number of Comorbidities		
Range	1–9	NA
Mean	3.36	NA
Marital Status		
Divorced	6	29%
Single	2	9%
Married/Domestic partner	8	38%
Widowed	4	19%
Separated	1	5%

## Data Availability

Data will not be made publicly available due to concerns for participant privacy. However data may be made available from the corresponding author at turrises@uncw.edu upon reasonable request and IRB approval.
